# Precision Augmentation of Medical Surge Capacity for Disaster Response

**DOI:** 10.1155/2020/5387043

**Published:** 2020-03-16

**Authors:** Weifeng Shen, Libing Jiang, Xiaojun He

**Affiliations:** ^1^Department of Emergency Medicine, The Second Affiliated Hospital, Zhejiang University School of Medicine, Hangzhou, China; ^2^Institute of Emergency Medicine, Zhejiang University, Hangzhou, China; ^3^Department of Chinese Journal of Emergency Medicine, The Second Affiliated Hospital, Zhejiang University School of Medicine, Hangzhou, China

## Abstract

**Background:**

In recent years, serious injuries associated with extreme climate, earthquakes, terrorism, and other natural and man-made disasters have occurred frequently throughout the world. A surge in medical demand that extends beyond local medical surge capacity in mass casualty incidents following major disasters is common. *Materials and Methods*. We reviewed and analyzed emergency medical rescue efforts after major disasters in recent years to elaborate the precision strategy of augmenting medical surge capacity for disaster response.

**Results:**

Precision augmentation of medical surge capacity for disaster response can be achieved through several measures. These include (1) release of internal capacity through precision launching or through upgrading the levels of response, (2) precision support for medical surge capacity from external efforts, (3) centralized response, and (4) altering standards of care. We should adopt precision augmentation of medical surge capacity according to the specific situation.

**Conclusions:**

Augmentation of medical surge capacity as a basic strategy can be used to achieve effective disaster response. In disaster response, due to the complexity of disaster medical capacity amplification, it is important to select the appropriate medical capacity strategy accurately according to the actual disaster situation.

## 1. Introduction

Large-scale catastrophic events can lead to mass casualty incidents, and catastrophic surge in medical demand often exceeds local medical surge capacity. Medical surge capacity refers to the capability of a medical system to cope with a sudden or sustained increase in demand for space, staff, supplies, and system operation through the rapid expansion of capacity [1, 2]. Medical surge capacity and capability for effectively expanding a healthcare system in a short period of time is an important aspect of disaster response [3, 4]. Augmentation of medical surge capacity as a basic strategy can be used to achieve an effective disaster response [5, 6]. Precision augmentation of medical surge capacity includes precision launching or increasing the level of emergency response to release internal surge capacity, precision support for medical surge capacity from external resources, centralized response, and altering standards of care [7]. Based on the theories and practices of disaster medical rescue in recent years, this study summarizes experiences of precision augmentation of medical surge capacity.

## 2. Precision Launching or Upgrading the Level of Response to Release Endogenous Medical Surge Capacity

Declaring a disaster to launch an emergency response is the primary mechanism for augmenting medical surge capacity according to a disaster plan [8, 9]. This is an immediately available response for an affected area. Based on the resilience of response, a local or regional response system releases endogenous surge capacity after launching an emergency response for a disaster. Endogenous augmentation of medical surge capacity refers to the increase in the medical surge capacity of a disaster response system through a self-buffer mechanism, which can also be called absorption capacity [10], which is inversely proportional to vulnerability and directly proportional to scalability. For example, in response to the Boston Marathon Bombing, due to the strong endogenous augmentation of medical surge capacity, the local medical response system had a favorable outcome with a low mortality rate of incident casualties [11]. Endogenous medical surge capacity mainly depends on the following aspects (see Table 1): (1) emergency response level, (2) structure of medical surge capacity, (3) number of key elements of response, (4) degree of matching in key elements of response, and (5) distribution of key elements of response in time and space.

### 2.1. Emergency Response Level

An emergency response level determines the level of surge capacity. Generally, emergency response levels can range from county level, to city level, to provincial level, to national level. These four emergency response levels can be fully integrated linked responses. The emergency response level can be dynamically increased to activate an appropriate response according to the actual disaster severity. In the initial stage of a major disaster, the magnitude of a disaster situation is unclear. Therefore, the disaster situation may be underestimated and the level of emergency response initiated may be lower than that required for the actual situation. With the gradual exposure of the magnitude of the disaster, the emergency response level needs to be adjusted according to the actual disaster situation. For example, in response to the 2008 Wenchuan earthquake in China, it was reflected on an increase in the level of initial response. Therefore, it is important to start an emergency response quickly in the event of a major disaster, and it is still more critical to accurately establish the emergency response level.

### 2.2. Structure of Medical Surge Capacity

Medical surge capacity in total includes healthcare facility-based surge capacity, public health-based surge capacity, and community-based surge capacity [12, 13]. The interaction and coordination of these three links of surge capacity are important. Moreover, it should be pointed out that these three links can be further subdivided. For instance, critical care capacity is an important component of healthcare facility-based surge capacity. Initial self-rescue and mutual rescue of communities are important efforts in the early stages of a disaster. In the specific stage of early disaster response, especially when professional rescue forces cannot arrive in time, local people's self-rescue and mutual rescue play a key role. After professional rescue forces arrive on the scene, self-rescue and mutual rescue of communities can continue to play a critical role in helping to identify dangerous zones and distribution zones of casualties, find water sources, and facilitate language communication. Citizens, volunteers, and social rescue forces play an irreplaceable role in saving the lives of victims after major disasters. Therefore, these three groups, which should not be ignored as they provide key supplemental help to professional rescue forces such as disaster medical assistance teams, are also an integral part of medical surge capacity. Due to the dynamic situation of disaster response, medical response capacity has a gradual release process. Moreover, the concept of a structural framework of medical response capacity is also in place. Hick proposed a progressive model that includes conventional capacity, contingency capacity, and crisis capacity [14]. Precise expansion of medical surge capacity should be based on a reasonable structure of medical surge capacity.

### 2.3. Essential Elements of Disaster Medical Response

Personnel, resources, space, and mechanisms, as the four basic elements, constitute medical surge capacity [15]. The magnitude of endogenous medical surge capacity released by a region is mainly related to the quantity of resources reserved for disaster response, emergency mobilization, and operation during a disaster. Within a reasonable range, endogenous medical surge capacity is quantitatively dependent on reserve resources, which is the theoretical basis for the endogenous expansion of medical surge capacity by activating reserve resources. An issue that requires further exploration is whether boundaries of quantitative dependencies exist between endogenous medical surge capacity and reserve resources. Is there a minimum quantity threshold or a maximum quantity threshold of the elements of disaster response? Minimum threshold refers to the lowest limit of the number of elements in an emergency medical response system. If the number of elements falls below the minimum threshold, a serious reduction in function or paralysis of an emergency medical response system may occur. Maximum threshold refers to the upper limit of the number of elements in an emergency medical response system. If the number of elements reaches or exceeds the maximum threshold, the medical surge capacity will not increase correspondingly or will not increase at all. The most probable cause is a bottleneck in the supporting system or elements. That is to say, once the integrated function is saturated, the contribution of additional elements to the medical surge capacity will be small or nonexistent, and adding more will even result in a counterproductive function. A theoretical framework of the minimum and maximum threshold of resources in medical response capacity can be used to explain the dependence of medical surge capacity on the availability of resources and how to use resources reasonably.

### 2.4. Degree of Matching in Essential Elements of Surge Capacity

Medical surge capacity is a system considered to be related not only to the quantity of response elements but also to the matching and combining of response elements. Hick proposed the COSTR model of the elements combination of medical surge capacity [16]. From the perspective of a seamless integration of prehospital and in-hospital response, medical surge capacity can be classified into medical rescue capacity, medical transport capacity, and hospital treatment capacity. In disaster medical response, if only part of the disaster medical response elements is available and in place, and other related elements are absent, it is difficult to ensure effective response. A well-coordinated response involving multiple resources is needed in a disaster. The emphasis should be on the timing combination of the disaster response elements, the continuity of prehospital care and hospital care in responding to a disaster [17], the coordination of medical response and public health response, the matching of the medical response system and the support system, and civil-military collaboration. In normal times, disaster response training and drills can promote the precise coordination of multiple response forces.

Finally, medical surge capacity should be dynamic and flexible in real time. Various medical surges show different curve shapes, with changes in a disaster process and spatial distribution of medical surges also present dynamically. Therefore, medical surge capacity deployment should change according to the time and space changes related to disaster medical surge. For instance, the epicenter of the 2008 Wenchuan earthquake was in Wenchuan County, China, and the most seriously damaged areas were not only the epicenter, but also the areas which were located where the distribution of fault zones was concentrated, namely, Beichuan, Mianzhu, Qingchuan, and other places. There was a deviation in the distribution of rescue forces in the early stage of the disaster response. After this problem was discovered, a dynamic spatial-temporal redistribution of medical surge capacity was carried out immediately. This was an important lesson learned from the response to the 2008 Wenchuan earthquake. Therefore, the precise expansion of medical surge capacity should also include the accurate spatiotemporal distribution of medical surge capacity in a disaster area.

In addition, strategies for augmenting medical surge capacity include medical institutions using reverse triage to create additional spaces or beds to receive the casualties in a disaster [18, 19].

## 3. Precision Support for Medical Surge Capacity from External Resources

Medical surge capacity can be increased through intrinsic and extrinsic mechanisms. For small-scale emergencies, the internal surge capacity released by the affected area is sufficient for dealing with medical surge. For major disasters, a significant number of resources from external support should be mobilized to the affected area as extrinsic surge capacity. Precision support for medical surge capacity from external resources is an important mechanism in disaster medical response. The following should be considered in exogenous precision augmentation of medical surge capacity: (1) An increase in external support does not mean medical surge capacity will be increased in a region. External supportive elements should meet the demand in the affected area. The key to achieving augmentation of medical surge capacity is the compatibility of external support. (2) External support for partial elements or resources of disaster medical response may consume or weaken other elements or resources in the affected area. For example, a large influx of rescue workers may accelerate the consumption of limited water and food in disaster areas. Similarly, a large influx of social vehicles may result in traffic congestion in a disaster area, delaying rescue forces from arriving and transferring mass casualties from the scene. (3) The spatial and temporal distribution and targeted supplementary external supportive medical forces should be considered. External support should be targeted to delivery according to the stage characteristics of medical surge and the state of disaster response. Available external resources need to be integrated with the internal elements of disaster response in the incident area to maximize medical surge capacity. There are two methods to ensure the organic integration of internal and external resources. The first method is that the internal and external elements of disaster response should be incorporated into the regional incident response framework in the incident area. Another method is that, if the regional incident response framework collapses after a devastating disaster, a new incident response framework should be constructed to integrate the internal and external elements of disaster response. These two methods are not contradictory. The key to providing external support is whether it is pertinent and effective [1, 20]. Which method of external support is more suitable for disaster response? If the response system in the incident area has not collapsed, the first method is applicable. The second method is appropriate in the event of a collapse of the response system in the incident area.

From the aspect of the quantity of resources, the available response resources = reserve resources + additional resources − consuming resources − unusable resources. Thus, the difference between medical surge and the sum of conventional capacity and endogenous augmentation of medical surge capacity is the exogenous augmentation required. The following should be considered regarding exogenous precision augmentation of medical surge capacity: (1) The requirement, difficulty, and possibility of achieving the exogenous augmentation of medical surge capacity combined with factors such as geography, climate, and traffic should be comprehensively evaluated based on the patterns, severity, urgency, and distribution characteristics of medical surge in the disaster area. It is also an important part of external support to identify priority response resources in order to expand or create more effective medical surge capacity as quickly as possible. (2) The baseline value of medical surge capacity and damage caused by a disaster as well as the availability of reserve resources can be used to measure the independent response capacity and duration of response in the affected area. The most seriously damaged and inadequate reserve resources in a disaster response are likely to be the critical link needed for the exogenous augmentation of medical surge capacity. (3) The contrastive relationship between medical surge and medical response capacity will be in a dynamic state (balanced, overwhelmed, crisis, and paralysis, respectively). Exogenous precision augmentation of medical surge capacity should also be dynamic according to the changes occurring during a disaster medical surge.

A major disaster can result in mass casualties and require a multiagency response. More importantly, significant interaction takes place between endogenous and exogenous augmentation of medical surge capacity for disaster response. The tiers of endogenous and exogenous surge capacity rely on the incident command system (ICS). In recent years, with the application of new technologies to emergency rescue such as fifth-generation mobile networks, unmanned aerial vehicles, and artificial intelligence information technology, ICS has played an increasingly effective role in precision augmentation of medical surge capacity. The main tiers of endogenous and exogenous surge capacity are coordinated response and time coincidence. The following should be included: (1) Tiers of response capacity: in addition to the type and quantity of elements, medical surge capacity depends on the matching degree in various elements of disaster medical response. Effective integration of different elements of response maximizes medical surge capacity. (2) Tiers of response time: it should minimize the interval from the time that startup is required for external support to the time that external support is received in an affected area. If external support action is delayed, autogenic initiating action is primarily required to be carried out and external support action will be launched only if medical surge is still overwhelmed in a disaster. For a major disaster, both autogenic and exogenous medical surge capacity amplifications need to be launched simultaneously. There may also be an extreme situation in which disaster-affected areas suffer a devastating blow, mainly relying on external support forces to form medical response capacity. Precision support for medical surge capacity from external resources needs to be fully integrated into the emergency medical response system.

## 4. Centralized Response to Maximize the Effectiveness of Limited Resources in a Disaster

Following the major earthquakes it has experienced in recent years, China developed the “four centralized response” principle for the efficient management of a major disaster. The core principle is to concentrate medical resources to respond to a large-scale medical surge in a state of limited resources. Traditionally, mass casualties have been diverted from the affected zone so as to achieve a balance between medical capacity and medical surge to the extent possible. However, a centralized response is different from this traditional approach. When a disaster medical surge has a clearly concentrated distribution, limited medical resources should be used in “one basket” to achieve the highest efficiency. When the distribution of medical surge in an affected area is not clear or is scattered, there are two methods for dealing with this. One method is to further divert the concentration of medical surge to alleviate the burden of the affected area. The other is to centralize the scattered medical needs so as to facilitate a centralized response. For these two methods, convenient transportation for mass casualties and the deployment of staff and resources are particularly required, and the establishment of an integrated air-ground transport system is particularly important. In recent years, the “four centralized response” principle, which is the centralized management of casualties, experts, resources, and treatment for medical rescue, has been developed on the basis of China's accumulated experiences with major disaster rescue [21]. Centralized response has become a principle with Chinese characteristics for responding to major disasters. The core of this principle is to centralize limited resources to achieve the maximum availability of capacity. This principle has been followed in China's major disaster response in recent years.

Precise implementation of a centralized response in disasters relies on two preconditions: (1) Operating condition. Disaster medical surge has not resulted in paralysis of a regional medical response system. The operating condition can help the concentration of resources and unified command to achieve a rapid and effective response as a whole. (2) Support condition. The lifeline system, such as the local or regional infrastructure, can be a bottleneck in the disaster response system. The lifeline system of a disaster area is never fundamentally destroyed and does not lose the ability to support basic disaster response. Remarkably, the advantages and disadvantages of a centralized response in a disaster are obvious. In special environments such as the block of casualties' transportation in disaster areas, it is not easy or may even be impossible to concentrate a response in a short time. Under these circumstances, if the principle of centralization is adhered to, the best time for rescue may be lost. Such a situation needs to be avoided.

## 5. Altering Standards of Care to Achieve the Relative Augmentation of Medical Surge Capacity in Crisis Response

As a fundamental issue, standards of care must be analyzed when resources are extremely scarce in crisis response. Ideally, altering standards of care might mean that constrained resources can be made available to rescue more casualties in a disaster. In theory, this strategy may be valid. Overviews of major disaster relief efforts have shown that standards of care for mass casualties in a disaster are associated with medical, ethical, or legal issues. Standards of care not only have an impact on the outcome of mass casualties but also can cause social concern. When resources are extremely scarce, reducing standards of care in a disaster may be regarded as “failure to provide proper care” for mass casualties in comparison with normal standards of care. Safety and quality issues in healthcare may be at risk of being impacted in a disaster. Therefore, it is necessary to balance the advantages and disadvantages of this strategy. After a thorough and detailed analysis of several major disaster relief experiences, we found that altering standards of care for mass casualties in disasters is a realistic issue, namely, that there might be “no other way.” The next issue, then, is whether “no other way” means optionally altering standards of care for mass casualties or following guidelines for a crisis response according to the actual situation of disaster response. At present, there are no generally accepted recommendations or guidelines for altering standards of care in disaster situations. At least it can be recognized that standards of care for mass casualties in disasters should be different from those in normal operating conditions. In the case of an extreme scarcity of resources, applying a damage control philosophy is also one of the options when conventional standards of care cannot be implemented. A damage control philosophy can be applied to reduce resource use and optimize surge capacity in a mass casualty response [22]. According to different disaster scenarios, standards of care for mass casualties in disasters may be categorized as conventional standards of care, emergency standards of care, and crisis standards of care [23]. In theory, properly reducing standards of care can be considered in order to reduce the average resource consumption for each casualty to maximize the efficiency of response when resources are extremely scarce in disaster response. The next question is how to judge the rationality of the altered standards of care. According to the current point of view, the decision to reduce standards of care for mass casualties must be made in situations involving a depletion of critical supplies or an overload of disaster medical surge. Altering standards of care as an alternative emergency mechanism may be implemented to adapt to a crisis response [24–26]. Altering standards of care, which can be called “choosing the lesser of two evils,” may be more effective and appropriate under the condition of severely constrained resources. However, this approach still requires further assessment of potential advantages and weaknesses. Remarkably, the reasonable and precise reduction of standards of care does not mean the absence of standards of care for mass casualties in a disaster situation. The key to providing an overall optimal response is the appropriate use of extremely scarce resources. To avoid confusion in implementing standards of care, there should be a return to normal standards of care as soon as possible once the disaster response system recovers from the crisis.

## 6. Conclusions

We emphasize the importance of precision augmentation of medical surge capacity in responding to catastrophic medical surge as quickly as possible. In this paper, we also note that precision augmentation of medical surge capacity should include precision launching or the upgrading of the levels of response to release internal surge capacity, precision support for medical surge capacity from external resources, centralized management to maximize the efficiency of response, and the altering of standards of care to achieve a relative expansion of medical surge capacity (see Table 2). In a disaster response, these strategies on the basis of launching a response level should be properly implemented according to the specific disaster scenario to achieve the best overall response effect.

## Figures and Tables

**Table 1:** Endogenous medical surge capacity mainly depends on the following aspects.

Serial number	Aspects
1	Emergency response level
2	Structure of medical surge capacity
3	Number of key elements of response
4	Degree of matching in key elements of response
5	Distribution of key elements of response in time and space

**Table 2:** Precision augmentation of medical surge capacity for disaster response.

Serial number	Measures
1	Release of internal capacity through precision launching or through upgrading the levels of response
2	Precision support for medical surge capacity from external efforts
3	Centralized response
4	Altering standards of care
